# Discourses of sustainability and imperial modes of food provision: agri-food-businesses and consumers in Germany

**DOI:** 10.1007/s10460-021-10269-z

**Published:** 2021-10-03

**Authors:** Steffen Hirth, Theresa Bürstmayr, Anke Strüver

**Affiliations:** 1 RCE Graz-Styria – Centre for Sustainable Social Transformation, University of Graz, Heinrichstraße 18, 8010 Graz, Austria; 2 Present Address: Alliance Manchester Business School, Sustainable Consumption Institute, The University of Manchester, Booth Street West, Manchester, M15 6PB UK; 3 Department of Geography and Regional Science, University of Graz, Heinrichstraße 36, 8010 Graz, Austria

**Keywords:** Imperial modes of living, Food sustainability, Discourse analysis, Governmentality, Everyday practices

## Abstract

It is widely accepted that overcoming the social-ecological crises we face requires major changes to the food system. However, opinions diverge on the question whether those ‘great efforts’ towards sustainability require *systemic* changes or merely systematic ones. Drawing upon Brand and Wissen’s concept of “imperial modes of living” (Rev Int Polit Econ 20:687–711, 2013; The imperial mode of living: everyday life and the ecological crisis of capitalism, Verso, London/New York, 2021), we ask whether the lively debates about sustainability and ‘ethical’ consumption among producers and consumers in Germany are far reaching enough to sufficiently reduce the imperial weight on the environment and other human and nonhuman animals. By combining discourse analysis of agri-food businesses’ sustainability reports with narrative consumer interviews, we examine understandings of sustainability in discourses concerning responsible food provision and shed light on how those discourses are inscribed in consumers’ everyday food practices. We adopt Ehgartner’s discursive frames of ‘consumer sovereignty’, ‘economic rationality’, and ‘stewardship’ to illustrate our findings, and add a fourth one of ‘legitimacy’. Constituting the conditions under which food-related themes become sustainability issues, these frames help businesses to (1) individualise the responsibility to enact changes, (2) tie efforts towards sustainability to financial profits, (3) subject people and nature to the combination of care and control, and (4) convey legitimacy through scientific authority. We discuss how these frames, mirrored in some consumer narratives, work to sideline deeper engagement with ecological sustainability and social justice, and how they brush aside the desires of some ostensibly ‘sovereign’ consumers to overcome imperial modes of food provision through much more far reaching, systemic changes. Finally, we reflect on possible paths towards a de-imperialised food system.

## Introduction

Food sustainability discourses have gained impetus from the goals of the Paris Agreement and the 2030 Agenda for Sustainable Development (SDG Knowledge Platform 2015) and it is widely accepted that overcoming social-ecological crises such as climate change and mass extinction (Ceballos et al. 2015) requires major changes to the food system. However, opinions seem to diverge on the question whether those ‘great efforts’ towards sustainability require *systemic* changes or merely systematic ones. We define the latter as efforts towards sustainability on an individual, corporate or personal level, whereas systemic changes would address structural factors of the political economy and society to lessen the burden of lifestyles on others and the environment by avoiding resource and energy use (e.g. through sufficiency, regulation, and practice change).

In public and academic debates on how ‘sustainable’ food practices can be achieved, plant-based, organic, local, seasonal, and fair commonly appear as the “grammars of good food” (Goodman and Jaworska 2020; see also Morgan 2010; Sage 2003). That consumers who are in a position to have a choice do in practice respond as ‘good citizens’ may give rise to hope, but the focus on these individuals obscures the fact that getting to the root of shifting unsustainable practices also requires forms of social-ecological citizenship in form of ethical producers, ethical provisioning networks, nonconsumption, and absolute reductions in both consumption and production (Fuchs et al. 2016; Goodman et al. 2010). The need for social-ecological transformations (Brand and Wissen 2017) is clearly recognised by major food policy institutions and academic consortia such as the EAT-Lancet commission (FAO 2006; IPCC 2019; Willett et al. 2019), but these commentators tend to emphasise diets, and thus consumer identities and choices, as an entry point to enact changes (Exner and Strüver 2020). A focus on diets entails risks not only by overlooking the socio-spatial relationalities of food production, processing, and provision, but also to overemphasise consumers’ abilities to enact the structural changes needed in the food system through changes in their purchasing activity.

In the wider context of environmental policy, Brand and Wissen (2013) identify a paradox between increased public awareness of social–ecological crises in the last few decades and relatively insignificant policy measures in response. They conceptualise this continuity of unsustainable practices as “imperial modes of living” (IML), pointing towards the political economy and ecology of capitalist developmental dynamics largely relying on “fossilist” patterns of production and consumption. Importantly, the particular context of food sustainability exhibits an analogous paradox by which consumers with normative orientations towards sustainability adopt diets with relatively high greenhouse gas emissions (Stieß and Hayn 2005; see also Wuppertal Institut 2008). Positioned within a broad range of social science literature on ethical consumerism and discourses on food sustainability, this paper applies the concept of IML to recent debates on ‘good food’ through an analysis of how producers and consumers allocate responsibilities for reconfiguring food provision towards sustainability. Critical food scholars, who have questioned tendencies to individualise the responsibility to enact positive changes to the food system, have compiled a rich base of literature both about food sustainability discourses, and about ethical consumers, but there is a lack of studies that combine both empirically through a balanced account of producers and consumers.

As part of the project Relational Geographies of Food, we complement the narrative consumer interviews introduced in earlier publications (Exner and Strüver 2020; Krüger and Strüver 2018) with a discourse analysis of the sustainability reports of agri-food-businesses and retailers, broadly subsumed under the term ‘producers’. We shed light on how understandings of food sustainability diverge or overlap in producer discourse and consumer narratives of ‘good food’, but also how those discourses are inscribed in consumers’ everyday food practices in Germany. We apply the concept of IML to the context of food provision in order to address barriers to sustainable food production and consumption and critically intervene in policy debates that underplay the opportunities of systemic changes.

In the following sections we first elaborate on what, with a nod to Brand and Wissen and their concept of IML, we call *imperial modes of food provision*. We then review the social scientific literature critically addressing the role of ‘the consumer’ as an agent of change towards food sustainability. The main findings section introduces Ehgartner’s discursive frames of ‘consumer sovereignty’, ‘economic rationality’, and ‘stewardship’ which we draw upon and adapt to our own case by formulating a fourth frame of ‘legitimacy’. In each subsection, we introduce one frame and subsequently present our findings by applying the discursive frames to the more specific topics that companies and consumers raise when they develop specific rationales (companies) or food practices (consumers) in the context of sustainability, and we show how the frames work to individualise sustainability problems and solutions. The subsequent section focuses entirely on those consumer narratives on ‘good food’ which indirectly or directly recognise the imperial character of current food provision. Beyond the individualised approaches in the preceding section, these comments express a desire for more profound, systemic changes to the food system than can be achieved through ‘ethical’ consumerism alone. The final discussion recalls how food sustainability discourses in form of the aforementioned frames work to systematically maintain imperial modes of living rather than help overcome them. We also point out some alternative frames, practices, and movements that would need to be brought up from the margins of food sustainability discourse in order to de-imperialise food provision and, thereby, take seriously the desire for systemic change some of our interviewees expressed.

## Imperial modes of living and ‘sustainable’ food provision

Ulrich Brand and Markus Wissen use the concept of IML to better understand the discrepancy between increased knowledge of the existential threats from various social-ecological crises and rather insignificant policy measures in response (Brand and Wissen 2013, 2017, 2021). The early 21st century has seen a “certain repoliticisation” expressed through the need to rebuild modern society away from fossil fuels, and whilst there is increasingly widespread knowledge of the multiple dimensions of this (local/global; social/economic/ecological) crisis, “these realisations have hardly led to the formulation, let alone the implementation, of far-reaching policies” (Brand and Wissen 2013, p. 688). IML draws on political ecology, regulation theory, hegemony, ecofeminism, and practice theory to explain why “fossilist” patterns of production and consumption persist. These patterns are “deeply rooted in everyday and institutional practices as well as societal orientations in the global North and imply a disproportionate claim on global resources, sinks and labour power” (Brand and Wissen 2013, p. 687).

By contrast, the term ‘sustainability’, which gained discursive thrust in the early 2000s as part of that repoliticisation, suggests that those societal deficits—the disproportionate claims to resources, sinks, and labour—can be overcome. Whilst there is also increased attention to ‘ethical’ consumption among middle and upper classes *in* the Global South (Gregson and Ferdous 2015; Hawkins 2015), by and large the phenomenon is still directed at Northern consumers providing ‘help’ *for* the South. It is important to acknowledge that sustainability may also be performed in a “quiet”, non-politicised form, without emphasis on ethical benefits for the environment or other people and as part of everyday practices of food self-provisioning and non-market exchange (e.g. in Eastern Europe or the Global South; Smith and Jehlička 2013; Feola et al. 2020). Analysing corporate communication, however, the focus here is on a relatively ‘loud’ articulation of sustainability. IML provides a framework for (re)thinking ‘ethical’ consumption and ‘sustainability’ as ideological tools to *maintain* unequal power relations, or at least slow down changes away from these.

We apply IML to the context of food sustainability. Whilst many actors involved in the food system “express interest in and support social justice goals, the incorporation of these goals into on-the-ground alternatives is often tenuous” (Allen 2008, p. 157). There is already a broad range of studies drawing on discourse approaches to point out various shortcomings of the food system (e.g. Lang and Barling 2012; Tomlinson 2013; Arcari 2017; Welch et al. 2018). Examining discourses of the food retail industry in the UK, Ehgartner (2018, 2020) finds that, rather than directly making consumers responsible for unsustainable practices, stakeholders use the dominant interpretation of the consumer as a “sovereign” to detract attention from the industry. As we elaborate on in the findings section, our study draws on Ehgartner’s discursive framework and applies it to the German case. Since this helps us to understand how unsustainable practices and inequalities are maintained, but lacks a link to the wider political economy, we use IML as a framework for our own research on food sustainability discourses. We shed light on, first, companies’ rationalities and actions towards food sustainability and, second, the ways in which these (and their ‘imperial’ characteristics) are appropriated or contested in the everyday practices of consumers. Our research suggests a corresponding discrepancy between the relatively high level of consumer awareness of the food system’s deficits, on the one hand, and the helplessly excessive resource use in everyday practices of production and consumption, on the other. Drawing on Brand and Wissen, we call this discrepancy *imperial modes of food provision*. We argue that, despite lively debates on social and ecological ‘sustainability’ and an increased range of products framed as ‘ethical’ consumption, dominant modes of food provision remain ‘imperial’ in the Global North.^1^

## Locating (ethical) consumption within the wider political economy

Our research resonates with a considerable body of literature from critical social sciences arguing (1) that consumers are (overly) responsibilised to mitigate social-ecological crises through consumption choices and (2) that a focus on consumption sidelines environmental problems and much needed systemic critique of producer–consumer and North–South relations.

When it comes to the (political) responsibility to enact changes, scholars have identified ‘the consumer’ as a powerful rhetoric figure who, as a ‘citizen consumer’ and analogous to a democratic system, ‘votes’ by choosing and purchasing products; through the aggregated activity of individuals, consumers are presented as impacting markets, for example, towards more ‘ethical’ consumption (Trentmann 2005; Sassatelli 2007; Barnett et al. 2011; Schwarzkopf 2011; Ehgartner 2018). Sociologists of (food) consumption have contested the individualising focus on choice by emphasising that consumption is often inconspicuously embedded in routinised practices (Warde 2005, 2016; Halkier 2010; Spurling et al. 2013).

More recent discussions of practice theory have critically reviewed the analytic separation between economically-connoted production and culturally-connoted consumption. There is a need for reconciliation by locating consumption within the wider context of political economy and elucidating practices within their systemic and material conditions for existence (Warde 2014; Evans 2020; see Welch et al. 2020 for an overview). This resonates with recent empirical work combining practice and materialist turns to conceptualise veganism not only as a dietary identity but more broadly as a food practice that includes production (Hirth 2021). In acknowledgment of Evans, who calls for “new conceptual vocabulary” (2020, p. 4) to think across the production-consumption dualism, this study combines consumers’ everyday food practices with food sustainability discourses and, by emphasising the imperial character of food provision, elucidates both against the background of the wider political economy.

This (re)orientation brings practice theory closer to other frameworks that provide critiques of individualism such as governmentality studies and Marxism. Both have traditionally provided fundamental, systemic challenges to the dominant social and economic order. Governmentality has been applied to outline the hegemony of the choice paradigm by explaining how subjects are controlled by means of direct conduct as well as indirect self-conduct in a neoliberal-capitalist order (Foucault 2008; see also Bröckling 2015; Nally 2011). According to Guthman (2007, p. 264) the focus on the freedom of consumer choices implies “a neoliberal anti-politics that devolves regulatory responsibility to consumers’ via their dietary choices”. Gunderson (2014, p. 109) regards “ethical” consumption as a “new layer of commodity fetishism” that reproduces systemic inequalities by conveying guilt-free consumption and, thereby, distracting from more profound systemic changes (see also Kalfagianni et al. 2020). Likewise, the concept of sustainability, conceived as a set of ‘fuzzy’ practices embedded and framed in a neoliberal capitalist order, is also accused of preventing radical changes (Swyngedouw 2007). Through these lenses, ordinary people who consent to their role as “a key agent of social change” (Barnett et al. 2011, p. 12) by trying to consume “ethically” or “alternatively” are seen as only mirroring the de-politicised neoliberal endorsement of private responsibility.

However, one of Barnett et al.’s main objectives is to dismantle these interpretations which derive from all too “’strong’ hypotheses about neoliberal subjects [and] might be in need of some revision” (2011, p. 20). They are concerned that scholars such as Guthman (2007) essentialise neoliberalism and treat it as a hegemonic force or social structure that cannot be changed. Guthman has, however, been rather self-critical towards her own arguments admitting that “it is difficult to know what something outside of neoliberalism might look like when all is seen as neoliberalism” (2008, p. 1181). Referring to Gibson-Graham’s (2006) “reading for difference, rather than dominance”, Harris provides a helpful discussion: Whilst *only* reading for dominance, he claims, carries “the potential to reinforce the alleged dominance of discursive neoliberalism, and thus to close down openings for constructive socio-environmental change” (2009, p. 61), an *additional* reading for difference is an approach that can recognise possibilities in alternative food practices, and that supports a politics of the possible (Harris 2009; see also Blay-Palmer et al. 2016 for a more recent account). Hence our project embraces both dominance and difference, conformity and resistance. Empirically, we do this by combining an analysis of large corporations with consumer narratives of everyday food practices. Theoretically, we pursue this by not only drawing on the self-conduct of individuals, which can involve both appropriation and resistance against discursive neoliberalism, but also on the imperial character of the political economy as a whole.

## Methods and data

The theoretical basis of our methodology is Foucault’s (1980) understanding of discourses as historically specific knowledge and power relations that produce reality and thus constitute and govern embodied subjects. In the context of this research, these linguistically and visually inscribed allocations of responsibility are conceived as power-laden attempts at defining and establishing ethical norms of food provision. Our qualitative methodology is based on a twofold approach, using discursive and semiotic interpretation for a combined analysis of agri-food businesses’ sustainability reports and narrative consumer interviews. The approach is focused on coding procedures applied to both documents and interviews, textual and visual material (Rose 2007). Drawing upon Grounded Theory (Glaser and Strauss 2017), discourse-analytic reconstructions were conducted using the software MAXQDA. This involved the systematic marking of text passages and images with descriptive terms (codes) which, by continuous comparison, were condensed to more abstract concepts (Diaz-Bone and Schneider 2010).

The first part of the research examined differences in how big agri-food businesses, retailers, and caterers operate within the semantic field of ‘good’, i.e. ‘responsible’ and ‘sustainable’ food and nutrition—how they conceptualise it, depict their own actions, and allocate responsibilities in pursuing it. The material examined included sustainability balance sheets and CSR reports, but also photos and figures on the companies’ websites and a few selected nongovernmental or critical media accounts of the companies’ activities (Table 1).

**Table 1:** Companies and the documents analysed, including the number of codes assigned to each document

Company	Type	Document type/title	Year	Number of codes
Alnatura	Organic supermarket chain and organic food producer	Sustainability Report	2013/14	277
		Website	2016	156
		WirtschaftsWoche critique	2014	10
McDonald’s	Fast food caterer/quick service restaurant	CR Report	2014	155
		CR Report	2015	315
		McDonald’s Germany in facts and figures—Supplement CR Report	2014	54
Monsanto	Agrochemical and biotechnology corporation	Sustainability Report	2014	673
		Annual Report	2015	77
		Myths and Facts	2016	74
		Website	2016	135
Nestlé	Food and drink processing corporation	Creating Shared Value Report	2014	794
		Progress Report	2014	381
		Oxfam Report: Behind the brands	2013	133
Rewe	Major conventional supermarket chain in Germany	Sustainability Report	2013/14	236
		Guidelines for sustainable development	2017	54
		CEO Statement	2016	27
		Oxfam critique about the Rainforest Alliance Label	2016	22

The selection criteria for the five companies are not based on either a representative image of the food industry as a whole or a focus on a specific type of company within it. However, with Gibson-Graham’s (2006) notion of “reading for difference” in mind, our sample is based on a wide variety of companies, regarding on the one hand the companies’ public visibility, awareness and ‘philosophy’, and on the other hand their underlying methods of production and nutrition that they promote and deploy. The companies range from seed provision to catering and retail, and they operate in different parts of the food system of provision. While Monsanto, Nestlé, and McDonald’s are multinational companies, Rewe and Alnatura operate almost exclusively in Germany and Austria. Furthermore, Alnatura exemplifies an alternative supermarket selling food certified as organic only, whereas McDonald’s and Rewe have a conventional offer with organic options. Initial inspections showed that the agrochemical and biotechnology company Monsanto^2^ (“Improving agriculture, improving lives”) and Alnatura (“Meaningful for humankind and earth”) try to convey similar moral intentions about providing food sustainably, even while the agricultural cultivation methods they represent—GMO vs. organic—are very different.

The second part of the research comprised 18 narrative interviews with consumers. Approached face-to-face near food retailers (conventional and organic supermarkets, discounters and a fast-food restaurant) in Hamburg and Berlin, the interviewees were recruited in both downscale and upscale residential areas as well as in fashionable commercial districts. The interviews were conducted directly on site, and the audio recordings were transcribed and analysed using MAXQDA.

Inspired by Schäfer and Völter (2005), the evaluation of the interviews involved exploring the relationship between discourse research and in-depth biographical case study research. In line with their approach, the appropriation and (re)production of discourses is an essential object of investigation. While conducting the interviews, narrative stimuli and subsequent immanent and exmanent questions were used to generate self-running narrative passages (Przyborski and Wohlrab-Sahr 2014). Two of the exmanent questions to our interview partners were, for example, what they understand by good and sustainable^3^ nutrition. Furthermore, we asked what it could mean to take responsibility in the field of nutrition and what paths to a good or sustainable food provision might look like.

In line with theoretical sampling (e.g. Auerbach and Silverstein 2003), relevant patterns of implicit and explicit use of discourses were reconstructed within a limited field of investigation. The sample of interviewees shows a wide variety regarding age, education, income, and gender. The first set of interviews showed little variation concerning other aspects that might have a crucial influence on food practices such as migration background or household types. Therefore, the second set of interviews addressed these shortcomings. Limitations of the sample remain in that, for example, all interviewees live in urban areas. However, rather than focusing on individual opinions or differences between groups, our approach is instead focused on “interpretative practices” and knowledge “repertoires” of interviewees (Talja 1999) and thus the variations present in public discourse. The purpose of the case studies was to reconstruct complex relationships between specific food-related identities, practices, and the (re)production of sustainability discourses.

## Systematic change: discursive frames of food sustainability

To illustrate our findings, we identify four discursive frames that recur as patterns in our data. We draw on Ehgartner’s (2020) discursive framework of food sustainability which includes ‘consumer sovereignty’, ‘economic rationality’, and ‘stewardship’. According to her, “these interpretative frames both represent and predetermine the conditions under which a food-related theme is dealt with as a matter and concern of sustainability” (2020, p. 476). In response to findings within our data, we adapt Ehgartner’s existing framework by adding a fourth frame about ‘legitimacy’. The following subsections combine producers’ rationalities about sustainability and their actually performed actions with consumer narratives, and both types of data are put in relation to the discursive frames. Table 2 exemplifies the four frames by help of producers’ statements on their own food sustainability commitments. An important discursive effect of these frames is that they sideline environmental and/or collective dimensions of sustainability. The superficiality of food sustainability discourses implied therein is a barrier to profound systemic changes that would de-imperialise food provision.

**Table 2:** Discursive Frames exemplified by companies' own statements on their food sustainability commitments

	Alnatura	McDonald’s	Monsanto	Nestlé	REWE
Consumer sovereignty	In order for organic farming to become more widespread, every year we are introducing to the market new organic products from Alnatura at an affordable price	We have introduced vegetarian burger variations or fruit and organic products in the Happy Meal as well as salt and fat reduced products	More and more people join the conversation about food, we have a responsibility to help make relevant information available to them	The nutritional composition of a product is becoming increasingly important to the consumer. Therefore, we have […] fundamentally revised the nutritional profile of our products. The focus was primarily on salt, saturated fatty acids and sugar	REWE Group intends to expand its range of more sustainable products and continuously expand and establish them in the mass market
Economic rationality	We want to operate our sites as energy-efficiently as possible. This means a continuous improvement process, both for existing and new stores	The effects of climate change such as droughts and floods are a growing challenge for global agriculture	The production of more food, more sustainably, requires the development of crops that can make better use of limited resources	Related to the race for land is the race for water. Water scarcity is already affecting almost one-fifth of the world’s population	REWE offers beverages in disposable packaging. It not only considers customer wishes [*consumer sovereignty*], but also takes advantage of potential savings opportunities in transport and handling
Stewardship	Organic farming is a culture of life. How we deal with nature is our responsibility. It is on this basis that we build our lives	McDonald's is committed to contribute to the integration of refugees in Germany	We have a responsibility to help ensure the world’s current population as well as future generations have enough of the right foods to eat	Nestlé invests in schools, and supports women to help them earn a better living	By opening new stores, communities benefit from infrastructure investments and taxes as well as the various social activities of employees, store managers and independent traders
Legitimacy	Some additives that are permitted under EU organic legislation are deliberately avoided	In order to continuously develop the applicable standards, we rely on close cooperation with research and science as well as our suppliers	In some cases, our Policy goes beyond what is required by law or what is customary in a region of the world	Nestlé is a higher performing company than the rest, having developed and published more policies aimed at tackling social and environmental risks within the supply chain	Conflicting goals are discussed with our experts as well as with external stakeholders. Thus, the company continues to develop its sustainability strategy and provides stimuli for new industry solutions

### Consumer sovereignty

This frame implies that producer efforts towards sustainable practices are conditional on a corresponding consumer demand. It involves references implying that sustainable development can only be achieved if consumers make corresponding choices, most commonly products conceived of as ‘ethical’ consumption.

In addition to Ehgartner’s account, we understand this frame as closely related to neoliberal forms of control and conduct. Foucault’s concept of governmentality implies that power—less and less centralised—works increasingly on and through private entities. Rather than through welfare policies of the government, social policy towards health and well-being—but here also sustainability—is sought by preventive appeals to individuals’ self-optimisation with the subject as an ‘entrepreneur of the self’ (Foucault 1988; 2008; Bröckling 2015). In the context of food provision, this frame thus constitutes consumers as what we call ‘providers of the self’. The transition away from imperial modes of food provision is thus rendered as a function of aggregated ‘good’ behaviour of individuals, particularly in moments of purchase, rather than an affair of institutional authorities or a sociopolitical issue in general.

Generally, the companies in our dataset emphasise the need to provide consumers with sustainable product options. They also seek consumer loyalty through communication and consumer participation. Nestlé, Rewe, McDonald’s and Alnatura focus on a strong consumer involvement in order to respond to their wishes and demands. Non-retailers such as Monsanto rather emphasise the importance of providing *information* on food sustainability. As a food processing company, Nestlé portrays health improvements in its product range as ‘important to the consumer’. As a result, Nestlé revised the recipes of products, focusing specifically on children’s products and products mainly consumed by children. Both portion sizes, as well as the products’ content of sugar, salt, or chemical additives, were reduced. This action can be framed as “choice editing”, a more recent communication strategy by which companies improve established products and portray the changes to the available choices as demanded by consumers (Ehgartner 2020). This illustrates that the depiction of consumers as ‘sovereigns’ is maintained even if companies make material amendments to products.

**Fig. 1:**
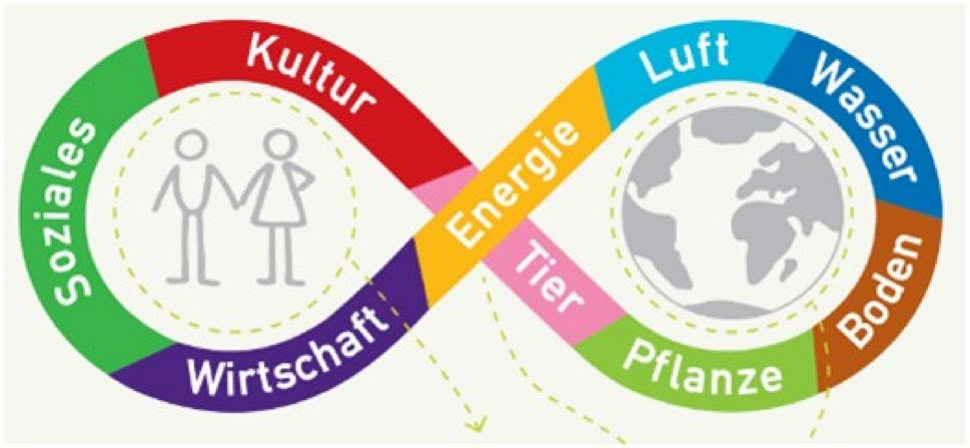
Alnatura’s lemniscate symbolising the entanglement of society and nature through social and cultural issues, animals, plants, soils, water, air, energy, and economy (https://www.alnatura.de/de-de/ueber-uns/nachhaltigkeit/nachhaltigkeit-bei-alnatura/)

Another characteristic of this frame are tensions between collective and individual agency and an implied bias towards the latter. Alnatura integrates a spiritual-cultural dimension to the classic three-pillar-concept of sustainability (i.e. ecological, social, and economic). By highlighting the importance of working “in harmony with nature, since, rather than humans, it is plants and animals who produce in agriculture”, the company decentres human agency in a posthumanist frame. To illustrate its understanding of sustainability it uses a symbol of eternity in which society and nature are entwined in mutually fruitful relations (see Fig. 1). Whilst this approach is depicted as the company’s approach to taking responsibility, it is also linked to *consumer sovereignty* by emphasising individual actions and awareness “because only the recognition of the effects of our behaviour enables us to recognise what is meaningful and to adjust our actions accordingly. […] Sustainable action follows from sustainable thinking. We want to promote this awareness, which each person can only achieve individually”. The holism at the horizon of Alnatura’s sustainability concept is thus in stark contrast to the company’s atomistic, individualised understanding of pathways towards that holism.

Interviewee statements that resonate with consumer sovereignty similarly emphasise choice and individual agency. Tf^4^ criticises (other) consumers’ high expectations to get, e.g., warm bread at all times, ignoring that this would necessarily result in food wasted. While she suggests consumers should put up with less choice to avoid food waste, she still identifies consumer choices as the crucial factor rather than holding businesses liable for their surpluses and disposal.

Af, a gardener and healer, can be characterised by a high degree of self-responsibility, and while she utters harsh critique of the current system, her choice of words shows individualising tendencies on two occasions. First, she asks “Where does it start? My responsibility. The profession I choose? […] Do I really work for Bayer? […] You must not speculate to such an extent with food or health!” Secondly, in the context of international migration, she speaks for open borders and against regulation which she regards as in the way of freedom, well-being, and peoples’ food security: “I believe if there was not so much regulation, things would have a chance to self-regulate”. By being against regulation and reflecting on individuals’ choices, she appropriates a language typical for neoliberal ideas of freedom. It is rather ironic, however, that neoliberal ideology is typically concerned with the ‘freedom’ of goods and companies, whereas the migration that Af would welcome is strongly regulated, bound to individuals’ socio-economic positions and the economic logic of ‘human capital’.

The addressed examples of food waste and open migration show that consumers typically want the world to be a ‘better’ place while their language mirrors the structural reasons for why it is not. Discourses in line with this frame exclude solutions beyond ‘improving’ consumers’ own choices. As consequence of conflating ‘the consumer’ with ‘sovereignty’, responsibility for (un)sustainable practices in the food system is individualised and diverted from businesses or policy makers.

### Economic rationality

Through this frame, actions towards ecologically or socially sustainable practices are constituted as desirable *on the condition that* they also appear “as ‘advantageous’ investments from a managerial and financial perspective” (Ehgartner 2020, p. 478). Our data on how businesses economically rationalise food sustainability ambitions or actions matches Ehgartner’s frame in three ways: resilience in the industry, cost-savings through eco-efficient production, and profitability through response to consumer trends.

Firstly, sane ecosystems and agricultural communities are fundamental to the very being of agri-food businesses and to society in general. Most companies emphasise the interdependencies through which people, businesses, and nature are linked. Monsanto, Nestlé, and Rewe concede that they could not exist without farmers. Nestlé also flags up problems in relation to that, such as regarding rural areas with an aging and decreasing population. Rewe mentions climate change as a threat to both yields and price stability. And Monsanto reminds us that “billions of people today and in the future are dependent on farmers”. Based on that insight, Monsanto tries to combine productivity with nature conservation, emphasising that this achievement also depends on others: “Now more than ever, we’re positioned to build upon initiatives, ideas and advances to help make a balanced meal more accessible to everyone, and doing it sustainably. Yet we know we can’t do it alone. We’re collaborating with others to tackle agriculture’s biggest challenges, helping to create a world of opportunity that can build a sustainable future for our business, our customers and our global community”.

Secondly, business stakeholders commonly emphasise the need to achieve sustainability through increased resource and energy efficiency. This includes Alnatura’s energy efficient buildings, Nestlé’s water-saving technologies, or Monsanto aiming to minimise resource and energy inputs and to maximise the output of crop varieties. In sum, a great deal of trust is put in technological improvements to achieve efficiency gains.

Thirdly, companies maintain profitability by responding to consumer trends*.* Traditionally relying heavily on beef (which, compared to other meats, has the highest environmental footprint; de Ruiter et al. 2017), McDonald’s has reacted to the popularisation of ‘plant-based’ diets by expanding its product range by adding meatless options. Whilst in its sustainability report McDonald’s does address meat’s problematic feed efficiency ratio, on the one hand, and the fact that more people become vegan, vegetarian, or flexitarian, on the other, it does not connect these topics semantically, treating the latter one as a mere question of lifestyle and identity without putting it in the context of (ecological) sustainability (see also Hirth 2021). This resonates with Ehgartner’s observation “that rather than *the problem of meat consumption* being discussed, it is the *opportunity of protein diversity*, which is mobilised” (2020, p. 480).

Interviewee positions reflect the presumption that companies change their offer in response to consumer trends such as vegan or organic foods. Rf and Sm mention the “vegan hype”. Having observed “the beginnings of vegan” as a trend, Rf suggests “it took about three years until it was integrated” into the offer of supermarkets. Um believes that increases in organic products and reductions of plastic bags happen “because the demand is there, and we can create the demand bottom-up”. He concludes “that power rather rests with consumerism. I think if we place ourselves in front of a supermarket and say “we don’t want this!” that will have less effect than if from now on we consume only in this way—this way they [the companies] will notice it much faster”.

Another interviewee holds consumers and their high standards accountable for companies’ unsustainable practices: “What is sustainable? That is all nonsense. […] If people have money, they consume and then it […] has to be beautiful, it has to be new. Who goes into an old McDonald’s, it must have nice, new tables, right?” (Im). Similarly, Rf has an apprehension that positive change will not happen if economic determinants are against it. After she criticises so-called influencers who promote foods virally on social media without questioning the chemical additives within, we ask her in which ways politicians are capable and responsible to change this situation. Rf admits that “I find it tricky. One must not forget the way things are. That sector [of viral ads in social media] exists and the economy is determined by supply and demand, and even if politicians say something, I don’t believe you can restrict that because there are always people who may put forward evidence, perhaps with a study, that those substances I criticised are maybe not that bad […] Ultimately, people have a responsibility to decide for themselves—What is good for me? What do I want? What can I afford?” Reducing the economy’s operating principles to supply and demand, this classical economic thought leads her in direct succession to the neoliberal emphasis on individual responsibility and the renouncement of regulation and state intervention. In sum, many interviewees share an understanding that companies cannot be expected to enact sustainable change of their own accord. If change happens, then because it is aligned with economic rationalities such as demand shifts that threaten profitability.

### Stewardship

As part of food sustainability, stakeholders commit themselves to specific values and moral integrity. This involves ‘caring’ for people and the environment (Kalfagianni et al. 2020) but also exercising benevolent control over both. As a frame, stewardship thus conveys an ethics of care in a generalised way of being ‘woke’ and pro-active about the social-ecological crises and challenges humanity faces. Most generally, many businesses emphasise their ‘philosophy’ by which they express an intellectual, ideational engagement with sustainability, including reflections on the ‘complexities’ and ‘interdependencies’ of production. More specifically, this involves responsible stewardship in the three contexts of global food security, farmer livelihoods, and measures against discrimination at work.

Firstly, one of the most prominent themes is protecting resources through efficient use. Monsanto presents increased efficiency as the only way to feed the growing world population without utilising resources beyond planetary boundaries. The company’s ‘care’ for global food security resonates with wider policy discourses on using “sustainable intensification” to double productivity by 2050 to “feed the 9 billion” (Lang and Barling 2012; Tomlinson 2013). Since Monsanto also uses this to promote its methods of biotechnology and “crop protection” through pesticide use, their approach of optimised efficiency to relieve pressure on soils and biodiversity, combined with productivity to feed everyone, also sits well with the economic rationality of maintained growth.

Feeding the world tends to overlap with a second theme of improving local farmer livelihoods in the Global South. Monsanto and Nestlé highlight the interdependency of their own economic success with the socio-economic well-being of communities in areas the companies operate in, which combines social with economic sustainability corresponding to stewardship and economic rationality. Beyond the general improvement of livelihoods, projects particularly focus on protecting children and empowering women. In the context of cocoa farming, Nestlé promises “easier access to training and support for women in our supply chain” and, thereby, “improves social conditions and reduces the risk of child labour”. Another example is the portrait of a Ghanaian maize farmer and shea-nut harvester who, according to Nestlé, has been able to improve her family’s living situation by increasing yields after participating in the company’s training courses in better farming methods (for an academic account of gender issues in cocoa supply chains see LeBaron and Gore 2020).

Thirdly, next to commitments to protect and educate children, there is an anti-discrimination theme by which businesses reassure pro-active measures against any form of discrimination against their employees along the lines of gender, race, and class. Nestlé is dedicated to gender equality particularly by increasing the number of women in leadership positions. Spatially, these commitments predominantly aim at the Global North, not least because farmers in the Global South are oftentimes only part of the supply chain, rather than employees. Deployment of topics such as equality and diversity are means through which the companies, above all McDonald’s, express that they care for their employees—in one case specifically for those who have migrated from the South. In the context of recent immigration to Germany from Syria and other conflict regions, McDonald’s emphasises that it provides employment, integration, and language training to refugees.

Interviewee positions match this frame by relating care to organic foods, fair trade products, nonhuman animals, local shop employees, farmers and rural culture: “I think that as a consumer one’s responsibility ranges from the environment to shop assistants or sales structures. Organic foods are not even that much better for myself but they derive from more reasonable conditions of production” (Cm). That this care tends to be conceived as a function of individual choice becomes particularly visible where interviewees explicitly refer to consumer behaviour and responsibility (e.g. Um, Fm, Yf).

### Legitimacy

To legitimise actions towards food sustainability, stakeholders seek scientific approval. As part of this frame, companies emphasise collaboration with partners—next to scientific experts, also non-governmental organisations—which secures their actions against external critique. That is, the authority of science, as executed through the continuous monitoring and evaluation of sustainability efforts and progress, endows businesses with credibility to be genuine and competent about their efforts. Legitimacy is sought in the three contexts of collaboration, monitoring, and a pairing of genuine goodwill with realistic recognition of imperfection.

Firstly, companies highlight the advantages of collaboration for achieving sustainability. Their efforts are aided by cooperation with partners and external experts, backed by their own or ‘independent’ research, in order to implement, maintain, and further develop responsible activities. Next to the composition of companies’ CSR reports, this involves reference to both widely-established as well as internally-developed labels and standards of production. In particular, Monsanto, McDonald’s, Rewe and Nestlé strongly emphasise the adoption of partly self-developed, partly established sustainability standards.^5^

Secondly, and interrelated with the reliance on external expertise, is the importance of continuous evaluation and control of sustainability parameters. Particularly in the context of the efficiency theme, this frame underpins actions of relative ‘improvement’ with positivist scientific authority and legitimacy. Ultimately, the quest for scientific rationality endows ‘improved’ production practices with credibility. Thereby, it is also entwined with the frames of *consumer sovereignty* and *economic rationality* since companies seek consumer approval and loyalty through ‘hard’ science and expert approval.

Thirdly, a keyword used by several companies is the need for “holistic” solutions. In combination with a world view of interdependency, these terms lead companies to remind of the immense ‘complexity’ of sustainability problems through which—endowed with legitimacy through scientific rationality and authority—they (re)assure that, despite their good will and efforts to tackle these problems, there is ‘no silver bullet’ to solve them. A realistic, genuine image of imperfection in relation to complex problems such as agricultural sustainability reduces the pressure to plan or enact changes beyond constant measurable and certified improvements, however small they may be.

The latter emphasis on sustainability’s complexity is mirrored by retired former corporate consultant Kf. She tries to live sustainably by buying regional, seasonal and organic food and only small amounts of meat, preferably from where farmers and employees are treated well. As a member of the Green Party, she is strongly aware of complex global interdependencies emphasising that there are no silver bullets for problems such as sustainability. “For ages, I am with the Greens, which is the only party that cares a bit [about sustainability], but first everybody needs to know one’s own mind. If everybody takes a little step, a lot is already achieved. And neither do they [the Greens] have the big solutions”. Elsewhere, Kf wonders how to get people to act more responsibly: “It is difficult—people don’t want it, they don’t want to grapple with such things, it’s too complicated. […] But this is a long-lasting problem—people who do not care and vote badly. Unfortunately, I have no solution how to instil into people that, one, it is complicated and, two, complicated solutions have to be developed for complex problems.” Whilst this emphasis on solutions fit for complexity conveys a move away from individual responsibility, her general choice of words very much focuses on the individual citizen-consumer and their (bad) choices.

Against the background of the unassailable complexity of sustainability problems and solutions, companies’ collaborations with external experts, measurable monitoring, and reliance on certified procedures appear all the more reasonable. The assurance that their efforts are—and cannot be anything but—a work-in-progress also takes weight off companies’ shoulders to quickly and fundamentally change their practices. Aligned with scientific rigour that is not known for pace, this frame pushes the obvious conclusion that “little steps” (Kf) might actually achieve *little impact* discretely into the background.

## Beyond individualising frames: desire for systemic change

The majority of consumers in our sample exhibit a deeply rooted frustration with the status quo of the food system—including the ones introduced in the previous section who exhibit tendencies to individualise the responsibility for change. While none of the producers address significant systemic changes, some consumers voice indirect and direct systemic critiques. Some of these interviewees do not directly call for the economic system to collapse, but they do critically specify societal problems that are inextricably entwined with capitalism. Others address the need to break down capitalist economic structures more explicitly. Their critique broadly involves the areas of the capitalist (food) system, neoliberal ideology, social inequalities within the Global North, and in North–South relations.

Taxi driver Im emphasises that “obviously, it is only the big corporations that can profit from [corporate bonds] because they are the only one’s creditworthy [laughs]. Personally, I find it’s a crazy world at the moment. I have nothing at all against such structures, but I am actually inclined against a few corporations in control of even the remotest corner of the world.” It is unclear whether with “such structures” he means capitalism or inequalities or large corporations, but the whole interview revolves around unequal corporate control of the food system which he is frustrated with, yet hopelessly regards as unassailable.

Out of concern for animal rights, Sm had already adopted a vegetarian diet as a child. He was also an active food saver. Today, he exclusively buys vegan products, whilst still eating animal-sourced foods whenever necessary to prevent them from going to waste. When it comes to questions of justice, he is very clear that changing diets alone will not be sufficient: “I do not believe that a transition towards a more just world can be achieved by maximising vegan and sustainable eating because, in a capitalist society, it cannot work like that since, regardless how much I advocate for people to become vegan, there is a too big industry, with too big interests, and far too much power, objecting to that. Thus, eating as vegan and sustainable as possible agrees better with my conscience, but […] I think real change will only happen when the economic structures change.”

Importantly, the critique is not confined to multinational corporations but also includes supposedly “alternative” actors. In teacher Fm’s view Alnatura’s selling practices are misleading. By example of the organic cheese he now avoids buying there because of the rind that is inedible, yet included in the price per kilo, he illustrates his impression that the company’s “maxim is no longer to sell good cheese which has its cost, but rather “We want to sell as much as possible at the highest margin!” […] I feel that good food and a mode of production that is too profit-oriented and efficiency-optimised are a bad match.” In discussing organic farmers’ association Demeter, computer scientist Cm, who tries to procure regional and seasonal foods at farmer’s markets, appreciates Demeter as a high agricultural standard and a counter to the industrial production system he rejects. However, while reflecting on his privileged situation, which enables him to purchase high-standard organic foods, he condemns the socio-economic conditions under which people acquire a high financial and moral position by making money (often at an environmental or social cost) and then taking ‘responsibility’ for the environment as a consumer without allowing others to do that. Similarly, medical student Sm identifies the prevalent individualisation of behavioural conduct as a red herring that deflects attention away from structures of inequality: “Medical science should much more focus on fostering health, rather than just treating sickness. And whilst fostering health *does* happen, it is always on an individual level. That is, ‘eat healthy foods’, ‘do not smoke’, ‘exercise’—things you would refer to as so-called ‘behavioural prevention’, attempts at changing people’s behaviour so they live healthier. And since food plays such a vital role in that context, I do not believe it is in proportion to what really makes us ill. There are also studies from England on the life expectancy of people with a different socio-economic position, that is, between rich and poor. The difference is more than 10 years, and that matters more than smoking—and nobody talks about that! It’s all just about ‘eat healthily’, but if you live in deprived conditions […], you just don’t have the possibility to eat healthily.”

A clerk on maternity leave, Tf takes offence at excess. Whilst on first sight this appears as a critique of consumer behaviour, she suggests a solution that draws on production: “Yes, I think we should create statistics displaying how many people produce how much of what—really not about what is consumed, but what is needed. [Interviewer: What is needed?] Exactly, and then we reduce correspondingly.” In sum, Tf may not explicitly speak against capitalism, but her critique of surplus production and her pleading for material sufficiency and planned food provision are not compatible with the imperatives of economic growth, corporate profits, and liberal markets.

Similarly, to the interviewer, doctor Yf first appears to individualise responsibility for a sustainable transition in one narrative, but she refuses to be construed that way and then clearly expresses a *shared* sense of it: “In every domain you can take responsibility, and you don’t have to wait until something happens politically or some legislation is put in place. Is it not the crux of our era that everybody is complaining about those at the top? Yes, but those up there only do what they get mirrored from the bottom. So, the responsibility is not with just one of us, we all have it… [Interviewer: …everybody for themselves, in the decisions we make] Everybody for themselves *and* for the world. It cannot work any differently.” More specifically, her relational understanding of global responsibility comes to the fore when she reflects on post-colonial North–South relations: “What I find really disastrous is that now all our jobless academic youngsters join NGOs to tell those countries how to do things right, and despite great potential, the people over there miss out again.” By claiming that relatively sound and ecological modes of production are being destroyed through modernisation in Togo, she exemplifies the need to protect certain practices against the (‘developmental’) dynamics of the economic system. Interestingly, she also apologises for “being so explicit about it”, anticipating other people’s disapproval of speaking frankly about post-colonial global injustices that entail a fundamental critique of Northern modes of living.

## Discussion and conclusion: the systematic maintenance of ‘imperial’ modes of food provision

According to Brand and Wissen (2013), the early 21st century has seen a ‘certain repoliticisation’ of unsustainable, yet normal, everyday practices, expressed through the need to rebuild modern society away from fossil fuels, or what they have coined ‘imperial modes of living’. We have applied IML to food sustainability discourses which, indeed, have become ubiquitous. A general need for change in the food system is a nearly uncontroversial claim today. Our data has not only shown that displaying their sustainability efforts “comes naturally” to all agri-food businesses, often in converging ways, but also that consumers unanimously express a deep frustration with, and often anxieties about, the status quo of food provision (see also Jackson 2015). We read this as an often implicit, sometimes explicit, perception that “normal” food practices are largely detrimental to the environment, other humans and nonhumans—in other words: imperial. Despite that ubiquity, increased societal awareness of crisis has not led to far-reaching policies to de-imperialise food provision in any materially and socially meaningful, i.e. systemic, way.

By shedding light on power-laden discourses on food sustainability, we have shown that, ironically, they can be forces that maintain imperial living systematically. Applying Ehgartner’s (2020) discursive frames of consumer sovereignty, economic rationality, and stewardship, extended by our own addition of the frame of legitimacy, has shown that there is significant overlap in producer discourses and consumer narratives regarding “good food”. Firstly, *consumer sovereignty* implies a predisposition that change is only justified if it is demand-led. Many consumer narratives mirror and accept that view by emphasising consumer behaviour as a driver of societal change. What is sidelined then are alternative perspectives that express the desire for changes by and to other economic and societal actors and structures. Secondly, *economic rationality* conjures novel technologies and efficiency implying that corporate sustainability efforts must also be profitable. In face of the “efficiency paradigm” (Zachmann 2012), we critically recall that behaviour in line with Jevon’s paradox—seeking “efficiency independently of limiting throughput” (Daly 2013, p. 24)—can help to maintain unsustainable, imperial practices, while conveying that, through constant ‘improvements’, a transition towards a better future would be not only under way, but also compatible with dominant economic rationalities (e.g. of growth). Thirdly, *stewardship* expresses care and responsibility towards others. However, it also bears the risk of “paternalistic” relationships to those being cared for (Puig de la Bellacasa 2010)—be it children, employees, farmers in the Global South, or nature—which, in turn, individualises the roots of problems associated with them. Finally, in the pursuit of *legitimacy,* companies draw on the authority of scientific experts, monitoring methods, and standards to seek approval for their chosen paths towards (more) sustainability. Relying on external expertise and certification while emphasising the ‘complexity’ of sustainability problems enables companies to convey an authentic sense of genuine goodwill and imperfection which may help them to develop strategies of “immunisation” against critics (Swyngedouw and Ernstson 2018); it is admitted that changes are time consuming, require scientific rigour, and even small steps towards sustainability are valorised. Particularly against the background of companies’ emphasis on efficiency, this frame underpins actions of relative ‘improvement’ with positivist scientific authority—thereby brushing aside the *remaining* impact of ‘improved’ practices in *absolute* terms (see also Fuchs et al. 2016).

Rather than significantly transforming food practices, the main effect of the four discursive frames works in favour of small steps of ostensibly systematic improvement, sometimes implemented by producers, yet always depicted as ‘chosen’ by consumers. This comes at the expense of any deeper, systemic engagement with ecological sustainability or social justice. Correspondingly, our sample of interviewees turns out as largely compliant with the imperative of ‘good food’ and their role as ‘sovereigns’ (e.g. eating no or moderate amounts of animal sourced foods, procuring organic, fair, regional, and seasonal foods). However, some of the interviewees, who equally care about ‘good food’, implicitly or explicitly express that, overall, food practices remain imperial and that, at the end of the day, they are not in the ‘sovereign’ position to implement significant changes, at least not in their role as consumers. While continuing to herald consumer sovereignty, companies’ food sustainability discourses anyway ignore the will of those ostensible ‘sovereigns’ who suggest systemic changes. In that guise, food sustainability discourse is the source of, rather than a challenge to, the systematic maintenance of imperial modes of food provision.

Really de-imperialising or “decolonizing food systems” (Figueroa-Helland et al. 2018) might instead require “carving spaces of possibility” that challenge the dominant, reductionist suggestions how to solve the various food crises (Moragues-Faus and Marsden 2017). First of all, this involves questioning the ideological basis of imperial living through alternative framings such as

- bringing down capitalism from its imperial heights framed as ‘the economy’ and reframing it as a mere set of contingent economic practices that has always already co-existed with a diversity of non-capitalist economic practices (Gibson-Graham 2006),
- understanding large ecological and social footprints as a symptom of systemic underdevelopment, particularly in so-called ‘developed’ countries (Ziai 2015),
- admitting that enacting sustainability by ‘good’ choices intrinsically fails because it paradoxically *requires* the actual production of ‘bad’ alternatives to decide against.

Beyond framings, there is a range of material-discursive practices that could help revitalise the food system if brought up from the margins of sustainability discourse. Firstly, movements of food sovereignty, agroecology, and indigenous revitalisation indicate that anti-colonial struggles persist in a world in which colonialism and imperialism are otherwise addressed as events of the past, if at all (Figueroa-Helland et al. 2018). Closely connected to struggles over access to land and ownership of the means of production is a rural revitalisation through small-scale, local and ecological farming in the Global South (IAASTD 2009) and beyond (Smaje 2020). Secondly, there is a need for appreciating and revitalising “endangered practices” such as repairing devices, mending clothes, foraging, and walking (Ehgartner and Hirth 2019) as well as tuning in to “quiet” or silenced forms of sustainable practices such as self-provisioning (Smith and Jehlička 2013; Feola et al. 2020). These practices are still more common, yet equally endangered, in the South and they are also part of global movements of social justice. Thirdly, advocating for veganism or flexitarianism as a dietary choice of individuals is insufficient as that bias foregoes the need to de-imperialise agriculture by reducing animal agriculture in absolute terms and by including novel agricultural movements such as vegan organic agriculture (Arcari 2017; Hirth 2021; Fuchs et al. 2016; Nobari 2021; Seymour and Utter 2021).

Today, the socio-ecology behind the COVID-19 pandemic (e.g. Settele et al. 2020) suggests that humanity and fellow beings are already losing Earth as a relatively “safe operating space” for our food systems (Rockström et al. 2009; Willett et al. 2019). Whilst the empirical data of this study precedes COVID-19, we find that the fundamental critique of the global economic and societal order some of our interviewees expressed is now becoming more salient to the general public highlighting serious deficits of an imperial food system. This crisis—and the wider one behind it—is yet another opportunity to respect the desires of allegedly ‘sovereign’ consumers towards systemic change.

## Abbreviations

CSR: Corporate social responsibility
GMO: Genetically modified organism
IML: Imperial modes of living

## Footnotes

1 And increasingly become ‘imperial’ in ‘developing’ parts of the South (see Brand and Wissen 2013); however, this is not within the empirical range of our research.

2 In 2018 Monsanto was acquired by Bayer AG as part of its crop science division.

3 We deliberately did not offer a definition for “good” or “sustainable” in order to reconstruct how interviewees (re)produced discourses on food. Whilst some interviewees eagerly took up or had already brought up the term “sustainable” on their own during narrative passages of the interview, others were unfamiliar with the term commenting only on their ideas of “good” nutrition (which nonetheless served as a proxy exhibiting sufficient overlap with sustainability).

4 To grant the interviewees’ anonymity we used codes instead of names. The first letter was assigned alphabetically (in the order the interviews were conducted). The second letter indicates the gender (f/m).

5 We distinguish this from Alnatura’s focus on the agricultural standards of organic production, rather than sustainability standards in general.
